# Selective SARS-CoV2 BA.2 escape of antibody Fc/Fc-receptor interactions

**DOI:** 10.1016/j.isci.2023.106582

**Published:** 2023-04-07

**Authors:** Yannic C. Bartsch, Deniz Cizmeci, Jaewon Kang, Hailong Gao, Wei Shi, Abishek Chandrashekar, Ai-ris Y. Collier, Bing Chen, Dan H. Barouch, Galit Alter

**Affiliations:** 1 Ragon Institute of MGH, MIT, and Harvard, Cambridge, MA 02139, USA; 2 Division of Molecular Medicine, Boston Children’s Hospital, Harvard Medical School, Boston, MA 02115, USA; 3 Beth Israel Deaconess Medical Center, Boston, MA 02215, USA

**Keywords:** Immunology, Virology

## Abstract

The number of mutations in the omicron (B.1.1.529) BA.1 variant of concern led to an unprecedented evasion of vaccine induced immunity. However, despite rise in global infections, severe disease did not increase proportionally and is likely linked to persistent recognition of BA.1 by T cells and non-neutralizing opsonophagocytic antibodies. Yet, the emergence of new sublineage BA.2, which is more transmissible than BA.1 despite relatively preserved neutralizing antibody responses, has raised the possibility that BA.2 may evade other vaccine-induced responses. Here, we comprehensively profiled the BNT162b2 vaccine-induced response to several VOCs, including omicron BA.1 and BA.2. While vaccine-induced immune responses were compromised against both omicron sublineages, vaccine-induced antibody isotype titers, and non-neutralizing Fc effector functions were attenuated to the omicron BA.2 spike compared to BA.1. Conversely, FcγR2a and FcγR2b binding was elevated to BA.2, albeit lower than BA.1 responses, potentially contributing to persistent protection against severity of disease.

## Highlights

• Systemic profiling of BA.1 and BA.2 specific antibodies at three doses BNT162b2
• mRNA vaccination elicits diminished BA.2 binding antibodies compared to BA.1
• Selective loss of BA.2 specific antibodies to leverage with FcγR3
• BA.2 specific antibodies poorly induced cytotoxicity and opsonophagocytosis

## Graphical abstract

**Figure:**
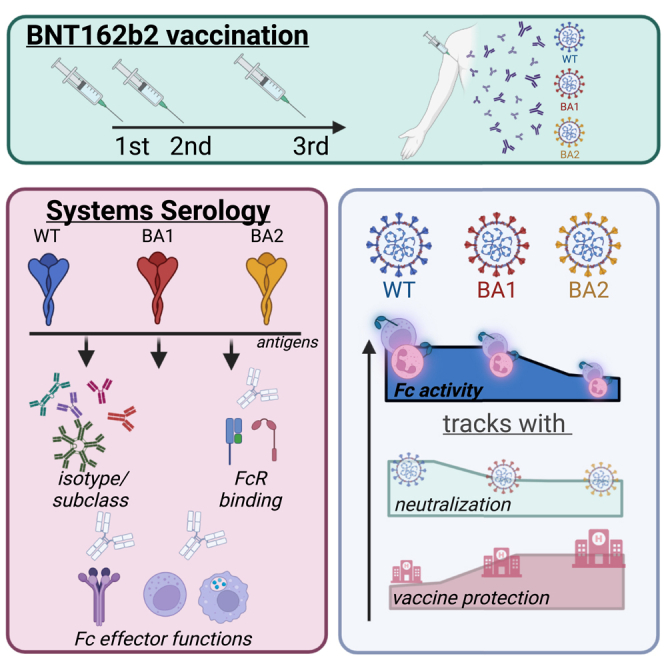

## Introduction

The rapid and perpetual evolution of SARS-CoV-2 continues to raise concerns for more pathogenic variants able to evade natural or vaccine-induced immune responses. With each wave, SARS-CoV-2 variants of concern (VOCs) have acquired mutations that increased infectivity, either via stabilization of the spike, enhanced binding to the angiotensin converting enzyme-2 (ACE-2), or via alterations in the viral fusion machinery.^1^^,^^2^^,^^3^ Evasion from neutralizing antibodies has progressed in parallel to this evolution due to the natural accumulation of mutations near or around sites involved in infection and fusion.^4^^,^^5^ The most recent VOCs, omicron, has exhibited the greatest evolutionary leap; acquiring 36 mutations, deletions, or insertions in the spike antigen, omicron is associated with significant evasion of vaccine-induced neutralizing antibodies and increased infectivity. However, despite this remarkable increase in transmissibility, the omicron BA.1 variant has exhibited overall lower pathogenicity and disease.^5^^,^^6^^,^^7^^,^^8^

Yet, a new omicron sublineage has emerged, the BA.2 lineage. While BA.2 spike lacks 16 of the alterations characteristic of BA.1, it has acquired 11 additional unique amino acid changes speculated to have increased BA.2 infectivity by 30% over BA.1.^9^ The BA.2 lineage has rapidly taken over the epidemic across Southeast Asia, Africa, and across Europe, and the America.^10^ Like the parental BA.1, BA.2 evades natural or waning vaccine-induced neutralizing antibodies equally and can be neutralized with boosting.^11^^,^^12^^,^^13^^,^^14^^,^^15^ While neutralizing, antibodies show minimally changed reactivity from BA.1 to BA.2, we speculated that other immune mechanisms may lose potency against BA.2, thus enabling this lineage to spread more efficiently.

In addition to neutralization, antibodies contribute to infection protection via their ability to use Fc-receptors to leverage the innate immune response and recruit phagocytes. Accumulating data are pointing to a role in Fc effector function in protection against severe COVID-19 infection, monoclonal therapeutic efficacy, and vaccine-mediated protection.^16^^,^^17^^,^^18^^,^^19^ Here, we aimed to define whether BA.2 evades these additional functions of the Pfizer BNT162b2 vaccine-induced humoral immune response at peak immunogenicity after the primary series, after eight months, as well as after a boost. We observed significant differences in vaccine-induced immunity to BA.1 and BA.2 marked by significantly lower antibody binding titers, reduced BA.2 FcγR3a and FcγR3b binding antibodies, and compromised Fc effector functions to BA.2. Thus, despite the near identical neutralizing antibody responses to BA.2, vaccine-induced Fc effector functions are selectively compromised to BA.2, potentially marking a weakened opsonophagocytic response to this virus that may be a key to attenuating transmission.

## Results

### Diminished isotype binding titers to BA.2

**Figure 1:**
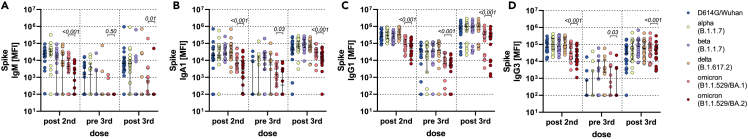
BNT162b2-induced antibody binding titer to different SARS-CoV-2 spike variants of concern Individuals received a three-dose regimen of the BNT162b2 vaccine. Samples were taken approx. 2 weeks after the second dose (post second, n = 18), before the third dose approx. 8 months after the second dose (pre third, n = 14) and approx. 2 weeks after the third dose (post third, n = 22). IgM (A), IgA1 (B), IgG1 (C) and IgG3 (D) binding titers to D614G (WT), alpha (B.1.1.7), beta (B.1.351), delta (B.1.617.2) variants of concern, and omicron (B.1.1.529) BA.1 and BA.2 subvariants spike were measured by Luminex. The average value of technical replicates is shown. The data were corrected for background and negative values were set to 100 for graphing purposes. A two-sided paired Wilcoxon test with a Benjamini-Hochberg post-test correcting for multiple comparisons was used to test for statistical differences between BA1 and BA2 titers, respectively. P-values are shown above each dataset. Horizontal lines indicate median and error bars the 95% confidence interval. See also Figure S2.

Despite nearly identical vaccine-induced neutralizing antibody responses to BA.1 and BA.2,^12^ the BA.2 sublineage exhibits a 30% increase in infectiousness and a potential increase in disease severity,^9^ calling into question whether this mutant may evade vaccine-induced immunity in a manner that is distinct from the original omicron BA.1 variant. Thus, we probed the ability of Pfizer BNT162b2 vaccine-induced antibodies to bind across VOCs, including the D614G (wild-type), alpha, beta, delta, and omicron BA.1 and BA.2 variants at peak immunogenicity (two weeks after the primary first and second vaccine), after eight months, and two weeks following a BNT162b2 boost (Figures 1, S1, and S2). Binding IgM, IgA, and IgG responses were probed. Robust IgM binding titers were observed across most VOCs after the primary series, albeit responses to BA.1 were lower and lowest to BA.2. IgM responses were rapidly lost over time and were not boosted against the beta, delta, or omicron spikes (Figure 1A). A similar profile was observed in the IgA response (Figure 1B), marked by robust IgA levels to the D614G wild-type, alpha, beta, and delta variant, but lower responses to the omicron BA.1 and even lower responses to the BA.2. Moreover, the responses waned over the first eight months proportionally to all variants, but most significantly for the omicron BA.2. Notably, all IgA responses boosted, albeit the omicron BA.1 and BA.2 responses never reached levels observed to the other VOCs. BA.2 IgA responses remained significantly lower to those observed to BA.1. Finally, IgG responses showed a similar profile to IgA, with lower IgG binding tiers to the omicron BA.1 and BA.2, with the lowest responses to the BA.2 spike (Figures 1C and 1D, Table 1). IgG1 and IgG3 levels waned markedly for all VOCs, but omicron binding IgG levels remained lowest compared to other VOCs. All VOCs IgG levels were boosted after the third dose of BNT162b2, although IgG1 to BA.2 did not boost proportionally (Table 2).

**Table 1:** Average fold reduction of indicated antibody features for the respective VOC spike proteins from post second dose to the pre third dose timepoint

	WT	Alpha	Beta	Delta	BA.1	BA.2
IgG1	952.7	832.9	504.5	669.0	297.5	300.9
IgA1	770.2	616.1	68.0	195.9	27.0	25.5
FcγR2a	2674.3	2347.6	1829.1	3945.7	644.2	66.1
FcγR2b	2726.2	1990.4	1554.3	1847.6	149.5	36.5
FcγR3a	1167.9	734.1	209.0	768.3	259.5	1388.6
FcγR3b	309.9	627.4	132.0	1478.1	12.4	40.2

**Table 2:** Average fold increase of indicated antibody features for the respective VOC spike proteins from pre third dose to the post third dose timepoint

	WT	Alpha	Beta	Delta	BA.1	BA.2
IgG1	486	584	512	560	213	187
IgA1	328	459	71	247	50	85
FcγR2a	3575	5388	4936	6658	2581	204
FcγR2b	6168	5725	5481	3392	1169	346
FcγR3a	1814	1365	713	1091	929	1216
FcγR3b	940	2426	642	2841	95	112

### Deficit FcγR3 binding to BA.2

**Figure 2:**
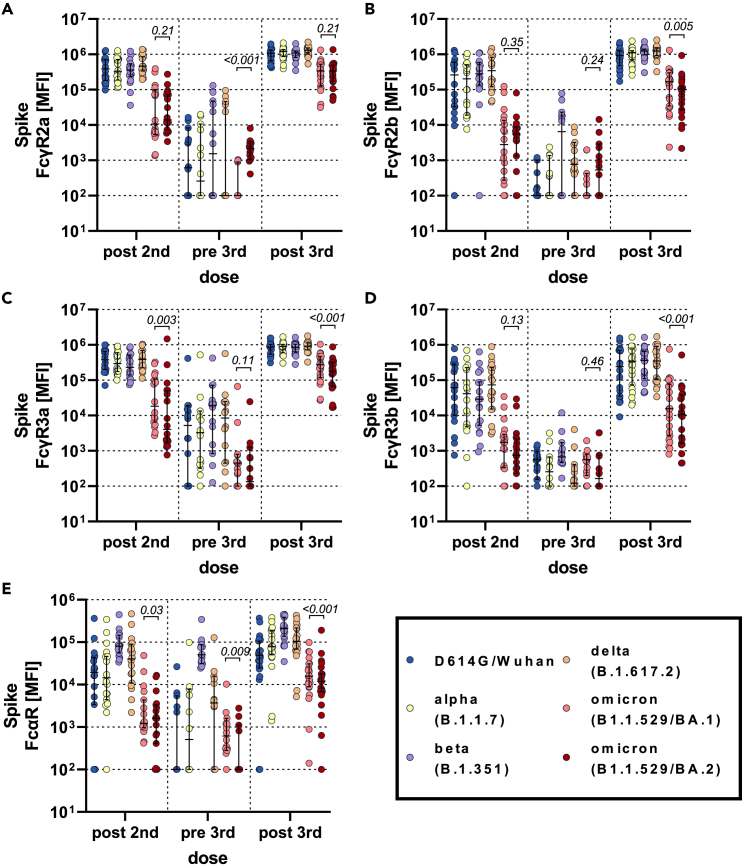
Fc-receptor binding antibody profiles across SARS-CoV-2 variants of concern Individuals received a three-dose regimen of the BNT162b2 vaccine. Samples were taken approx. 2 weeks after the second dose (post second, n = 18), before the third dose approx. 8 months after the second dose (pre third, n = 14) and approx. 2 weeks after the third dose (post third, n = 22). Binding to FcγR2a (A), FcγR2b (B), FcγR3a (C), FcγR3b (D), and FcαR (E) of D614G (WT), alpha (B.1.1.7), beta (B.1.351), delta (B.1.617.2) variants of concern, and omicron (B.1.1.529) BA.1 and BA.2 subvariants spike specific antibodies were measured by Luminex. The average value of technical replicates is shown. The data were corrected for background and negative values were set to 100 for graphing purposes. A two-sided paired Wilcoxon test with a Benjamini-Hochberg post-test correcting for multiple comparisons was used to test for statistical differences between BA1 and BA2 titers, respectively. P-values are shown above each dataset. Horizontal lines indicate median and error bars the 95% confidence interval. See also Figures S3 and S4.

Given the diminished binding profiles to the BA.2 variant, we next aimed to investigate whether this compromised recognition of the BA.2 spike also translated to reduced Fcγ-receptor (FcγR) binding. We profiled BNT162b2 vaccine-induced spike-specific FcγR binding across the four low-affinity human FcγRs involved in driving non-neutralizing Fc-effector functions (Figures 2 and S3). Interestingly, binding to the opsonophagocytic activating FcγR2a showed diminished omicron BA.1 and BA.2 reactivity compared to the D614G/wild-type, alpha, beta, and delta variants (Figure 2A). Attenuated omicron BA.1 and BA.2 reactivity persisted with waning and after the boost; however, FcγR2a binding to the BA.2 was not different at post-vaccine timepoints (prime and boost) and remained higher than BA.1 at the waning time point. Conversely, the inhibitory FcγR2b was not different across the omicron sublineages at peak primary immunogenicity, BA.2 FcγR2b binding was higher than BA.1 at the waning time point, and BA.2 FcγR2b binding was lower than BA.1 binding after the boost, with omicron reactivity being lower than the other VOCs across all time points (Figure 2B). Finally, vaccine-induced spike-binding antibodies to the cytotoxicity FcγR3a and neutrophil specific FcγR3b and the IgA binding FcαR showed a similar pattern of lower overall omicron binding responses after the peak primary immune response, although BA.2 exhibited the lowest FcγR3 binding (Figures 2C–2E). At waning time points both omicron responses elicited substantially lower FcγR3a and FcαR binding than for other VOCs. After boosting, all VOC-specific FcγR3b binding responses increased, although omicron and beta responses increased to a lesser degree (Table 1). Likewise, Fc-receptor binding was highly correlated with antibody titer only at the post-boost time point, highlighting the importance of the third dose in driving coordinated humoral immune responses (Figure S4). Thus, these data point to conserved activating opsonophagocytic BA.2 specific FcγR2a binding, but marked loss of FcγR2b, FcγR3a, and FcγR3b binding to the BA.2 sublineage.

### Vaccine-induced antibodies poorly leverage Fc-effector functions against the BA.2 sublineage spike

**Figure 3:**
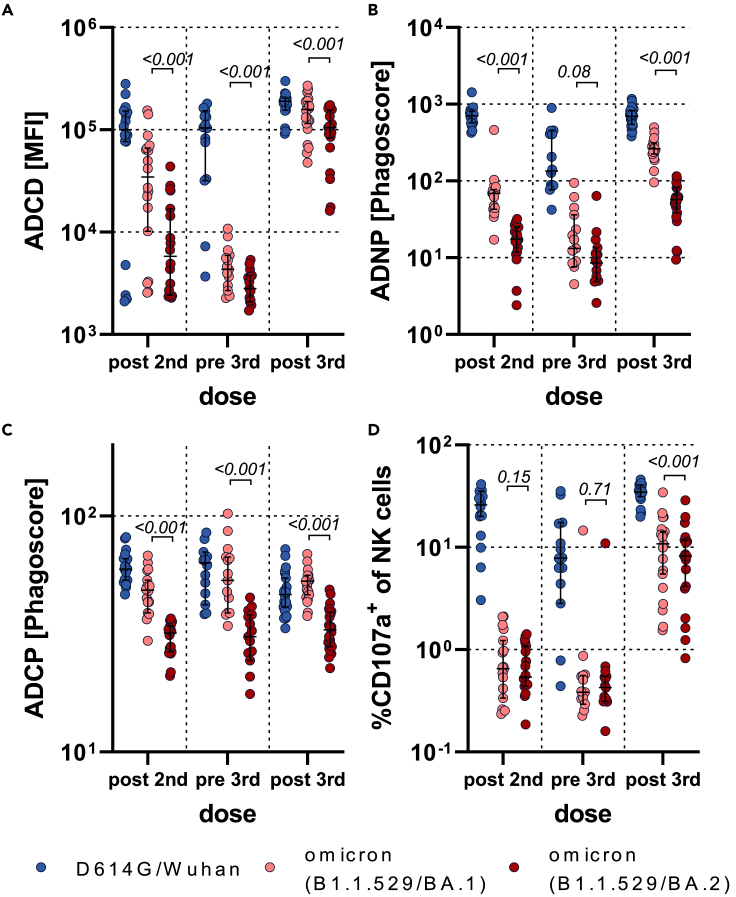
Fc functionality of BNT162b2-induced antibodies is superior to BA.1 compared to BA.2 antigen Antibody-dependent complement deposition (ADCD) (A), Neutrophil phagocytosis (ADNP) (B), Cellular monocyte phagocytosis (ADCP) (C), or NK cell activation (marked by CD107a expression) (D) of D614G, omicron (B.1.1.529) BA.1 or BA.2 specific antibodies was analyzed in BNT162b2 recipients at approx. 2 weeks after the second dose (post second, n = 18), before the third dose approx. 8 months after the second dose (pre third, n = 14) and approx. 2 weeks after the third dose (post third, n = 22). The average value of two donors is shown for ADNP and ADNKA or of two technical replicates for ADCD and ADCP. The data were corrected for background and negative values were set to 1 for graphing purposes. A two-sided paired Wilcoxon test with a Benjamini-Hochberg post-test correcting for multiple comparisons was used to test for statistical differences between BA1 and BA2 titers, respectively. P-values are shown above each dataset. Horizontal lines indicate median and error bars the 95% confidence interval. See also Figures S5–S8.

Given the observed compromised FcR binding profiles, we aimed to define whether vaccine-induced antibodies could leverage antibody effector functions. At peak immunogenicity after the primary series, BNT162b2-induced spike-specific antibodies drove substantially compromised omicron BA.1 antibody-dependent complement deposition (ADCD), neutrophil phagocytosis (ADNP), monocyte phagocytosis (ADCP), and natural killer (NK) cell activation (ADNKA) compared to the D614G spike (Figures 3 and S5). BNT162b2-induced spike-specific functional activity was further compromised to the omicron BA.2 spike. Fc effector activity declined proportionally over time across all of the spike variants, albeit the BA.2 activity remained the lowest. Boosting increased Fc effector activity to all spike variants, although omicron spike responses never reached those of the D614G/Wuhan response, and BA.2 remained the lowest. These data suggest that non-neutralizing antibody effector functions are severely compromised at all timepoints for the BA.2 omicron sublineage, likely resulting in compromised opsonization, FcR activation, and viral clearance to help mitigate disease and transmission.

### Multivariate signatures of defective immunity to BA.2

**Figure 4:**
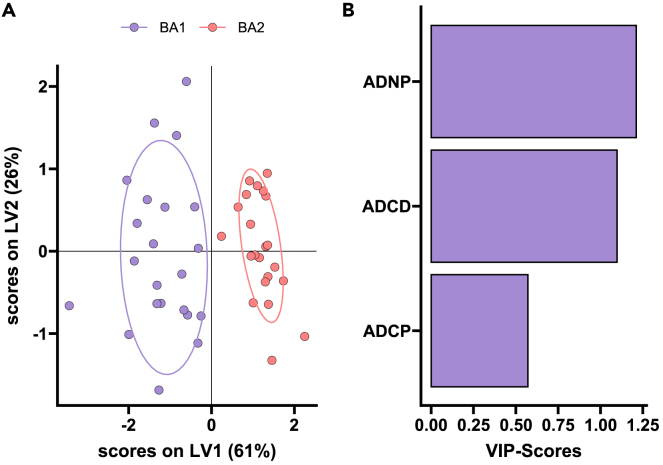
BNT162b2 induces distinct BA.1 and BA.2 specific responses A machine learning model was built using BA.1 and BA.2 spike specific features in BNT162b2 vaccinated individuals post third dose (n = 22). (A) A minimal set of LASSO selected features was used to discriminate between humoral responses in a PLS-DA analysis. Each point represents an individual’s humoral response for BA.1 (purple) and BA.2 (red). (B) Selected features were ordered according to their variable importance in projection (VIP) score (purple = enriched for BA.1 antigen).

To finally gain a detailed understanding of the fundamental differences in the BNT162b2-induced response to BA.1 and BA.2 that may result in less protection against BA.2, we generated a multivariate partial least squares-discriminant analysis (PLS-DA) model across the two spike antigens at peak immunogenicity after the third dose (Figure 4). Perfect separation was noted across the BA.1 and BA.2 responses (Figure 4A), marked by elevated Fc functionality exclusively to the BA.1 lineage. Notably, BA.1-specific immune profiles exhibited a selective enrichment of spike-specific ADCD, ADCP, and ADNP (Figure 4B). Beyond the overall compromised vaccine-induced immune response to omicron BA.1, vaccine-induced immune responses are further reduced to BA.2, marked by more limited functional IgG/FcγR responses that may lead to poorer control and clearance of this sublineage.

## Discussion

Early phase 3 vaccine trial immune correlates analyses, at a time when DG14G/Wuhan and the alpha variant dominated the global pandemic, pointed to the importance of neutralizing antibodies in protection against COVID-19.^20^ However, the emergence of the beta, delta, and now omicron VOCs, all of which significantly evade neutralizing antibody responses but do not cause more disease among vaccinated populations,^5^^,^^6^^,^^7^^,^^8^ has raised the possibility that alternative vaccine-induced immune responses may be key to protection. Both vaccine-induced T cell and non-neutralizing antibody responses have shown enhanced resilience to VOCs and have been proposed to contribute to attenuated disease against these evolving neutralizing antibody-escaping variants. However, the emergence of the omicron BA.2 sublineage has begun to challenge this hypothesis. Specifically, BA.2 exhibits enhanced transmissibility compared to the BA.1 lineage, despite only 1.5-fold reduced neutralizing antibody activity to BA.2.^12^^,^^13^ Thus, reduced neutralizing antibody cannot account for enhanced transmissibility of this new sublineage of omicron. Given our increasing appreciation for a role for non-neutralizing Fc effector antibody responses in protection against natural severe disease,^21^^,^^22^ in monoclonal therapeutic activity,^16^^,^^17^^,^^18^^,^^19^ and in vaccine mediated protection,^23^ here we profiled the Pfizer BNT162b2 functional humoral immune response across VOCs including BA.1 and BA.2. As expected, vaccine induced non-neutralizing antibody responses were lower to omicron variants but were most profoundly diminished against the BA.2 spike antigen. However, selective Fc-receptor binding deficits were noted, marked by maintained/superior FcγR2a/b binding, but reduced FcγR3a/3b binding to BA.2 compared to BA.1. Linked to reduced neutrophil opsonophagocytic activity (Figure S6), these data point to a selective loss of IgA/neutrophil functional mucosal immunity that may be a key to protection against transmission, but maintenance of FcγR2a/b that may continue to confer protection against disease.

Omicron BA.1 included 36 mutations in the spike antigen that significantly evaded vaccine-induced and monoclonal therapeutic recognition.^5^ Similarly, to the loss of neutralizing antibody responses, Moderna mRNA1273, Pfizer BNT162b2, and CoronaVac vaccine induced immune responses lost recognition of receptor binding domain (RBD) but maintained robust recognition and non-neutralizing antibody responses to the BA.1 spike antigen.^24^ Conversely, BA.2 includes 11 additional spike mutations that appear to further compromise antibody binding to the spike, resulting in compromised Fc-receptor binding and significantly lower non-neutralizing antibody responses to this new sublineage. Whether these additional 11 mutations result in disruption of immune complex density, or create geometric changes in the spike, precluding the formation of robust immune complexes and binding to particular Fc-receptors remains incompletely understood but could provide key insights for the design of next generation vaccine-antigen inserts to maximize the induction of highly functional antibodies that, in addition to neutralization, may be a key to protection against transmission. Thus, it will be critical to map the precise functional epitope footprints of protective antibodies to guide vaccine design in the future.

Compared to our previous findings of deficits in omicron-specific VOCs-specific Fc effector activity in mRNA vaccine recipients, the data presented here point to a more significant loss of BA.2 specific Fc effector function.^25^ Furthermore, direct comparison of BNT162b2 immunity to BA.1 and BA.2 pointed to selective defects in the BA.2-specific response, marked by a global loss of FcγR dependent effector responses, and linked to a specific loss of FcγR2b, FcγR3a, and FcγR3b binding. Moreover, we observed a titer-independent loss of complement fixing activity, potentially related to the loss of IgM binding to omicron, as well as to geometric differences in IgG recognition of the mutated omicron sublineage spikes that may potentially preclude complement binding, the key for cytotoxic destruction, induction of opsonophagocytosis, and T cell immunity.^26^^,^^27^ Moreover, coupled to the quantitative loss of opsonophagocytic and cytotoxic functions, these data point to a unique axis of immunity that may be lost to BA.2. Specifically, neutrophils and monocytes respond to both IgG1/3 immune complexes via antibody binding to FcγR2a and FcγR3b, IgM and IgA-formed immune complexes via the constitutive expression of FcμR^28^ or FcαR,^29^^,^^30^ and via complement receptors,^31^ enabling phagocytes to rapidly clear immune complexes both systemically, as well as at mucosal membranes. In contrast to this compromised Fc effector activity, neutralizing antibody responses were similar across omicron sublineages.^12^^,^^13^ Additionally, while neutralization is also highly dependent on antibody titers, this activity is traditionally reported in a titer-uncorrected manner. However, upon titer correction of Fc effector data, we observed a strong relationship between antibody binding differences (titer) and ADCP, ADNP, and ADNKA, but enhanced complement fixing defects even after titer correction. These data suggest that reduced BA.2 cross-reactivity induced binding defects may contribute to reduced Fc effector mediated control of this omicron sublineage, further compounded by compromised complement activity. On a per-antibody level, no difference in BA.1 or BA.2 specific antibodies that elicit ADNP, ADCP or ADNKA were observed. Although, BA.1 specific antibodies were more prone to induce Fc-receptor independent complement deposition (ADCD) than BA.2 antibodies on the post second and third dose time points (Figure S7). Thus, the global loss Fc effector activity against BA.2 may point to a selective deficit in humoral BA.2 specific immunity related to both quantitative and qualitative changes in antibody recognition of the VOCs.

Emerging vaccine efficacy data point to preserved protection against BA.2.^32^ However, why similar levels of neutralization permit BA.2 to infect more efficiently than BA.1 may be related to changes in the viral spike antigen^33^ and compromised alternative antibody effector functions that may work synergistically with neutralization to promote full control of the virus at the mucosal barrier. Both opsonophagocytic and cytotoxic functions at the mucosal barrier are linked to protection against several infectious diseases including *Streptococcus pneumoniae*,^34^ Respiratory Syncytial Virus,^35^ Influenza,^36^^,^^37^ etc. Moreover, Fc effector functions may potentiate the immune-protective role of neutralizing antibodies via collaboration between the constant domain (Fc) and antigen binding (Fab) domain of antibodies. Thus, it is plausible that the selective loss of collaborative Fc activity may diminish the sustained protection against BA.2 despite relatively preserved neutralization, pointing to opsonophagocytosis and complement cytotoxicity, coupled to neutralization, as potential critical correlate of immunity against BA.2 and other VOCs.

### Limitations of the study

While this study did not explore differences in antibody effector function to additional omicron sublineages (BA.2.12.1, BA.2.75, BA.4, or BA.5), nuanced binding has been observed across the newer sublineages that likely account for further evasion of Fc effector function. Moreover, this study did not explore functional humoral immunity to BA.2 following additional vaccine platforms, yet increased omicron sublineage transmission appears to occur globally in a vaccine platform independent manner, even after boosting,^38^ suggesting that promoting more wild-type-specific spike immunity may be insufficient to drive robust antibody effector functions against these rapidly evolving variants. However, while boosting with omicron spike led to a marginal increase in neutralization across VOCs compared to the wild-type antigen, these studies did not take into consideration the critical nature of VOCs breadth of binding for shaping Fc effector function. Thus, future efforts focusing on the importance of VOCs based vaccination may unravel the critical nature of vaccine-induced breadth on shaping additional antibody effector responses that may be a key to protection against severity of disease, rather than simple blockade of transmission. Linked to future quantitative assessments of Fc effector levels, rather than qualitative measurements presented here, thresholds of protective immunity may be defined to guide future boosting recommendations.

## STAR★Methods

### Key resources table

**Table:** 

REAGENT or RESOURCE	SOURCE	IDENTIFIER
**Antibodies**		

anti-human IgG1	SouthernBiotech	Cat# 9052-09, RRID:AB_2796621
anti-human IgG3	SouthernBiotech	Cat# 9210-09, RRID:AB_2796701
anti-human IgM	SouthernBiotech	Cat# 9020-09, RRID:AB_2796577
anti-human IgA1	SouthernBiotech	Cat# 9130-09, RRID:AB_2796656
anti-human CD66b	BioLegend	Cat# 305112, RRID:AB_2563294
anti-guinea pig C3	MP Biomedicals	Cat# 0855385, RRID:AB_2334913
anti-human CD107a	BioLegend	Cat# 328634, RRID:AB_2563851
anti-human CD56	BD Biosciences	Cat# 335791, RRID:AB_399970
anti-human CD3	BioLegend	Cat# 300426, RRID:AB_830755
anti-human MIP-1β	BD Biosciences	Cat# 562900, RRID:AB_2737877
anti-human IFNg	BD Biosciences	Cat# 562900, RRID:AB_2737877
anti-human CD32 (FcγRIIA)	Bio X Cell	Cat# BE0224, RRID:AB_2687707
anti-human CD16 (FcγRIII)	Bio-Rad	Cat# MCA1193GA, RRID:AB_324304

**Chemicals, peptides, and recombinant proteins**		

SARS-CoV-2 Spike S1+S2 (D614G) trimer Protein (ECD, His tag)	SinoBiological	Cat: 40589-V08H8, YP_009724390.1
SARS-CoV-2 B.1.1.7 Spike S1+S2 trimer Protein (ECD, His tag)	SinoBiological	Cat: 40589-V08H12, YP_009724390.1
SARS-CoV-2 B.1.351 Spike S1+S2 trimer Protein (ECD, His tag)	SinoBiological	Cat: 40589-V08H13, YP_009724390.1
SARS-CoV-2 B.1.617.2 Spike S1+S2 trimer Protein (ECD, His tag)	SinoBiological	Cat: 40589-V08H10, YP_009724390.1
Human Fc receptors	Produced at the Duke Human Vaccine Institute	NA
FIX&Perm Cell Permeabilization Kit	Thermo Fisher Scientific	Cat: GAS004
EDC	Thermo Fisher Scientific	Cat: A35391
Sulfo-NHS	Thermo Fisher Scientific	Cat: A39269
EZ-link™ Sulfo-NHS-LC-LC-Biotin	Thermo Fisher Scientific	Cat: A35358
FluoSphere™ NeutrAvidin™ conjugated beads	Thermo Fisher Scientific	Cat: F8776 and F8775
Lyophilized Guinea Pig complement	Cedarlane	Cat: CL4051
Gelatin veronal buffer	Sigma-Aldrich	Cat: G6514
GolgiStop	BD Biosciences	Cat: E1483
4% Paraformaldehyde	SantaCruz Biotechnology	Cat: Sc-281692
Ammonium-Chloride-Potassium (ACK) buffer	VWR	Cat: 10128-802

**Critical commercial assays**		

RosetteSep Human NK Cell Enrichment Cocktail	Stem Cell Technologies	Cat: 15065

**Experimental models: Cell lines**		

THP-1 cells	ATCC	CAT#: TIB-202 RRID: CVCL_0006
Expi293F cells	Thermo Fisher Scientific	Cat: A14527, RRID:CVCL_D615

**Recombinant DNA**		

Mammalian cell expression vector pCMV-IRES-puro	Codex BioSolutions, Inc	

**Software and algorithms**		

Intellicyt ForeCyt Software	Sartorious	https://intellicyt.com/products/software/
R programming language	https://www.r-project.org/	Version 4.0.1
R Studio	https://www.rstudio.com/	Version 1.3.1093
Prism 9	Graph Pad	Version 9.3.1

### Resource availability

#### Lead contact

Further information and requests for resources and reagents should be directed to and will be fulfilled by the lead contact, Galit Alter (ragonsystemserology@mgh.harvard.edu).

#### Materials availability

This study did not generate new unique reagents.

### Experimental model and subject details

#### Study population

Plasma samples from a total of 24 BNT162b2 vaccinated individuals (median age: 34 years, range: 23-69 years, 88 % female) were obtained from a specimen biorepository at Beth Israel Deaconess Medical Center (BIDMC). Participants received three doses of 30 μg BNT162b2. The first doses were given approx. 21 days apart as per manufacturers recommendation. The third dose was given a median 254 days (range 248-258 days) after the second dose (Figure S1). Samples for all three timepoints were available for 13 individuals while for three included individuals only the post 2^nd^ and post 3^rd^ dose timepoint and for one individual the pre- and post 3^rd^ timepoint was available. Additionally, two individuals with only the post 2^nd^ dose and four individuals with the post 3^rd^ dose timepoint were included in the analysis. The individuals that did not have a first or second sample were all age and gender matched and did not have any reported co-morbidities. All participants provided informed consent prior to enrollment into the study. No participant reported or had serological evidence (Nucleocapsid-specific antibody titer) of previous SARS-CoV-2 infection, received other COVID-19 vaccines, or immunosuppressive medications. This study was overseen and approved by the BIDMC Institutional Review Board (#2020P000361) and the MassGeneral Institutional Review Board (#2021P002628).

### Method details

#### Antigens and biotinylation

Spike protein antigens for the D614G wildtype, alpha (B.1.1.7), beta (B.1.351), and delta (B.1.617.2) VOCs were obtained from Sino-Biologicals. Omicron (B.1.1.529) BA1 and BA2 Spikes were produced in house.^39^ All antigens were produced in mammalian HEK293 cells. A strep-tag for purification was added to the C-terminus of the Omicron Spikes, whereas all other Spike variants had a His-tag at the C-terminus. All Spike antigens were expressed in the HexaPro (S-2P) form to stabilize the prefusion state of the protein. For functional assays all antigens were biotinylated using an NHS-Sulfo-LC-LC kit according to the manufacturer’s instruction (Thermo Fisher Scientific). Excessive biotin was removed by size exclusion chromatography using Zeba-Spin desalting columns (7kDa cutoff, Thermo Fisher Scientific).

#### IgG subclass, isotype and FcγR binding

Antigen specific antibody subclass, isotypes, and FcγR binding was analyzed in technical replicates by Luminex technology. Antigens were coupled to Luminex beads (Luminex Corp, TX, USA) by carbodiimide-NHS ester-chemistry with an individual region per antigen. Coupled beads were incubated with diluted plasma sample (1:100 for IgG3, IgM and IgA1, 1:500 for IgG1, FcαR and 1:2,000 for FcγR probing) for two hours at room temperature in 384 well plates (Greiner Bio-One, Germany). Unbound antibodies were washed away and subclasses, isotypes were detected with a respective PE-conjugated antibody (anti-human IgG1 (Cat# 9052-09, RRID:AB_2796621) , IgG3 (Cat# 9210-09, RRID:AB_2796701), IgM (Cat# 9020-09, RRID:AB_2796577) or IgA1 (Cat# 9130-09, RRID:AB_2796656) all SouthernBiotech, AL, USA) at a 1:100 dilution. For the analysis of FcγR binding PE-Streptavidin (Agilent Technologies, CA, USA) was coupled to recombinant and biotinylated human FcγR2a, FcγR2b, FcγR3a, FcγR3b or FcαR protein (Duke Human Vaccine Institute Protein Production Facility). Coupled FcR were used as a secondary probe at a 1:1000 dilution. After one hour incubation, excessive secondary reagent was washed away and the relative antibody concentration per antigen determined on an iQue analyzer (IntelliCyt). Each sample was analyzed in duplicates.

#### Antibody-dependent-neutrophil-phagocytosis (ADNP)

Phagocytosis score of primary human neutrophils was determined as described before.^30^ Antigens were biotinylated with NHS-Sulfo-LC-LC kit according to the manufacturer’s instruction (Thermo Fisher). Excessive biotin was removed by size exclusion chromatography using Zeba-Spin desalting columns (7 kDa cutoff, Thermo Fisher). Biotinylated antigens were coupled to fluorescent neutravidin beads (Thermo Fisher) and incubated with 1:10 diluted plasma. Primary cells were derived from Ammonium-Chloride-Potassium (ACK) buffer lysed whole blood from healthy donors and incubated with immune complexes for one hour at 37°C. For the Fc-receptor blocking experiments, isolated neutrophils were pre-incubated with 5 μg/ml of FcγR2a (CD32A, clone IV.3, Bio X Cell Cat# BE0224, RRID:AB_2687707) and FcγR3 (CD16, clone: LNK16, Bio-Rad, RRID:AB_324304) five minutes prior to addition of neutrophils to the immune complexes. Neutrophils were stained for surface CD66b (BioLegend Cat# 305112, RRID:AB_2563294) expression, fixed with 4% para-formaldehyde, and analyzed an iQue analyzer (IntelliCyt) (Figure S8).

#### ADCD assay

For the complement deposition assay,^40^ biotinylated antigens were coupled to FluoSphere NeutrAvidin beads (Thermo Fisher Scientific) and incubated with 10 μl 1:10 diluted plasma samples for two hours at 37°C. After non-specific antibodies were washed away, immune-complexes were incubated with guinea pig complement in GVB++ buffer (Sigma-Aldrich) for 20 minutes at 37°C. Complement reaction was stopped with EDTA-containing phosphate-buffered saline (15mM) and C3 deposition on beads was stained with a 1:100 diluted anti-guinea pig C3-FITC antibody (MP Biomedicals, Cat# 0855385, RRID:AB_2334913) and analyzed on an iQue analyzer (Intellicyt). Each sample was analyzed in duplicates (Figure S8).

#### ADCP assay

Biotinylated antigens were coupled to FluoSphere NeutrAvidin beads (Thermo Fisher Scientific) and incubated with 10 μl 1:10 diluted plasma for two hours at 37°C to form immune complexes.^41^ THP-1 monocytes (American Type Culture Collection) were added to the beads, incubated for 16 hours at 37°C, washed and fixed with 4% paraformaldehyde. Samples were analyzed on an iQue analyzer (Intellicyt). Each sample was analyzed in duplicates. (Figure S8).

#### ADNKA assay

To determine antibody-dependent NK cell activation, ELISA plates (MaxiSorp, Thermo Fisher Scientific) were coated with respective antigen for two hours at room temperature and then blocked with 5% bovine serum albumin (BSA, Sigma-Aldrich) overnight. 50 μl 1:20 diluted plasma sample was added to the wells and incubated overnight at 4°C. NK cells were isolated from buffy coats from healthy donors using the RosetteSep NK cell enrichment kit and SepMate50 tubes (STEMCELL Technologies). Isolated NK cells were stimulated with recombinant human interleukin-15 (1ng/ml, STEMCELL Technologies) at 37°C overnight. The next day, NK cells were added to the washed ELISA plate and incubated together with anti-human CD107a BV605 (BioLegend Cat# 328634, RRID:AB_2563851), brefeldin A (Sigma-Aldrich), and monensin (BD Biosciences) for five hours at 37°C. Cells were then stained on the surface with CD56-PE-Cy7 (BD Biosciences Cat# 335791, RRID:AB_399970), and 1:800 CD3-APC-Cy7 (BioLegend Cat# 300426, RRID:AB_830755). Cells were fixed and permeabilized with FIX & PERM Cell Permeabilization Kit (Thermo Fisher Scientific), and afterwards stained for intracellular markers using anti-human-MIP-1β BV421 (BD Biosciences Cat# 562900, RRID:AB_2737877) and anti-human IFN-γ PE (BD Biosciences Cat# 554701, RRID:AB_395518). NK cells were defined as CD3-CD16^+^CD56^+^ and frequencies of degranulated (CD107a+), IFN-γ+ and MIP-1β+ NK cells determined on an iQue analyzer (Intellicyt).^42^ Each sample was tested with NK cells from three different donors (biological triplicate) (Figure S8).

### Quantification and statistical analysis

#### Computational analysis

A multivariate classification model was built to discriminate humoral profiles between BA.1 and BA.2 specific antibodies. Prior to analysis, all data were normalized using z-scoring. Feature selection was performed using least absolute shrinkage and selection operator (LASSO). Classification and visualization were performed using partial least square discriminant analysis (PLS-DA). Selected features were ordered according to their Variable Importance in Projection (VIP) score and the first two latent variables (LVs) of the PLS-DA model were used to visualize the samples. Visual separation of BA.1 and BA.2 responses in the PLS-DA was observed when all or only LASSO selected features were used. These analyses were performed using R package “ropls” version 1.20.0^43^ and “glmnet” version 4.0.2^44^ and the systemseRology R package (v.1.1) (https://github.com/LoosC/systemsseRology).

#### Statistical analysis

If not stated otherwise, we assumed non-normal distributions and plots were generated and statistical differences between two groups were calculated in Graph Pad Prism V.9. A paired Wilcoxon test with a Benjamini-Hochberg post-test correcting for multiple comparisons was used to test for statistical differences between BA.1 and BA.2 features at the different timepoints.

## Data Availability

All relevant data is reported in this paper. This paper does not report original code. Any additional information or raw data will be shared by the lead contact upon reasonable request.
