# Supplementary material for: Selective SARS-CoV2 BA.2 escape of antibody Fc/Fc-receptor interactions
Source: iScience. 2023 Apr 7;26(5):106582. doi: 10.1016/j.isci.2023.106582 (PMC10079316; doi:10.1016/j.isci.2023.106582)
Supplement: Document S1. Figures S1–S8 [file mmc1.pdf]

iScience, Volume *26*

Supplemental information

Selective SARS-CoV2 BA.2 escape

# of antibody Fc/Fc-receptor interactions

Yannic C. Bartsch, Deniz Cizmeci, Jaewon Kang, Hailong Gao, Wei Shi, Abishek Chandrashekar, Ai-ris Y. Collier, Bing Chen, Dan H. Barouch, and Galit Alter

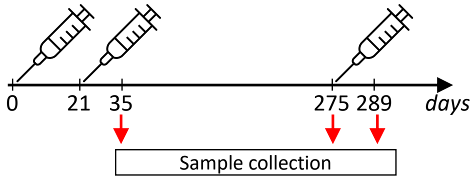

Figure S1 Vaccination and sample collection timeline. Related to Results and STAR Methods. Participants received the first two doses BNT162b2 approx. 21 days apart and the third dose approx. 275 days after the first dose as indicated by syringe icons. Samples were taken approx. on day 35 (two weeks after the second dose), on day 275 (before the third dose) and on day 289 (2 weeks after the third dose) as indicated by red arrows.

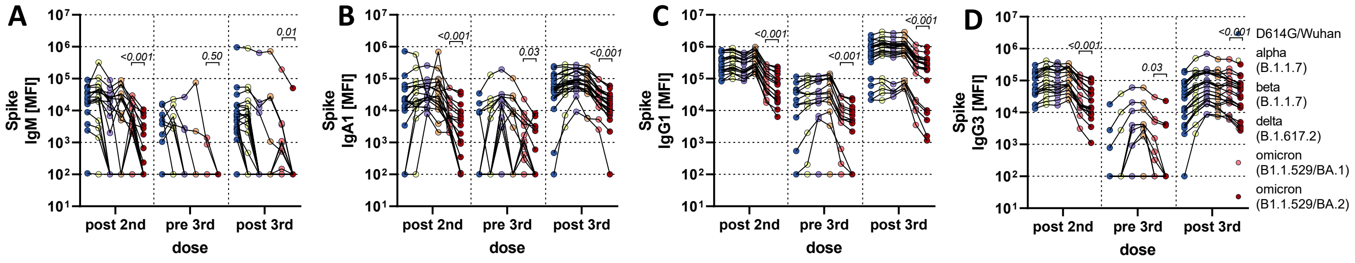

Figure S2: BNT162b2 induced antibody binding titer to different SARS-CoV-2 Spike variants of concern. Data as in Figure 1. Lines connect data from same individuals.

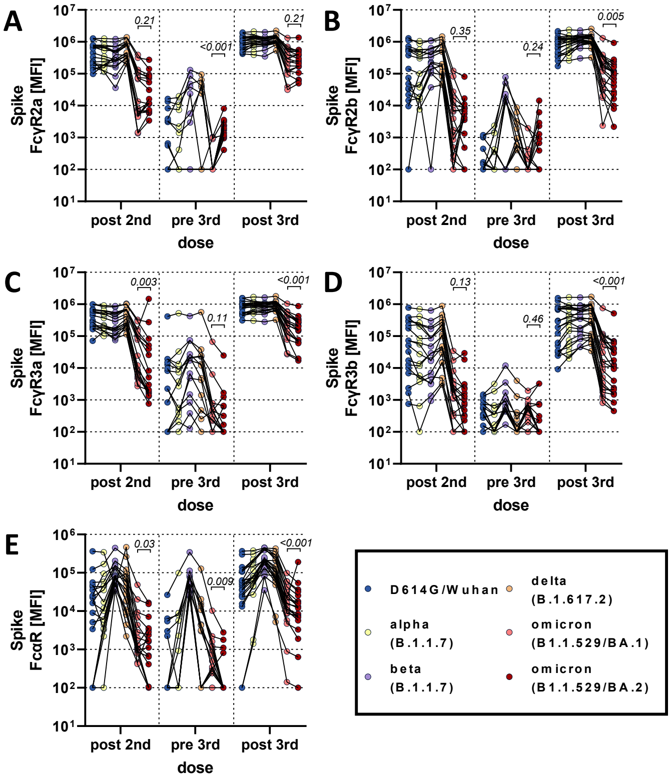

Figure S3. Fcγ-receptor binding antibody profiles across SARS-CoV-2 variants of concern Data as in Figure 2. Lines connect data from same individuals.

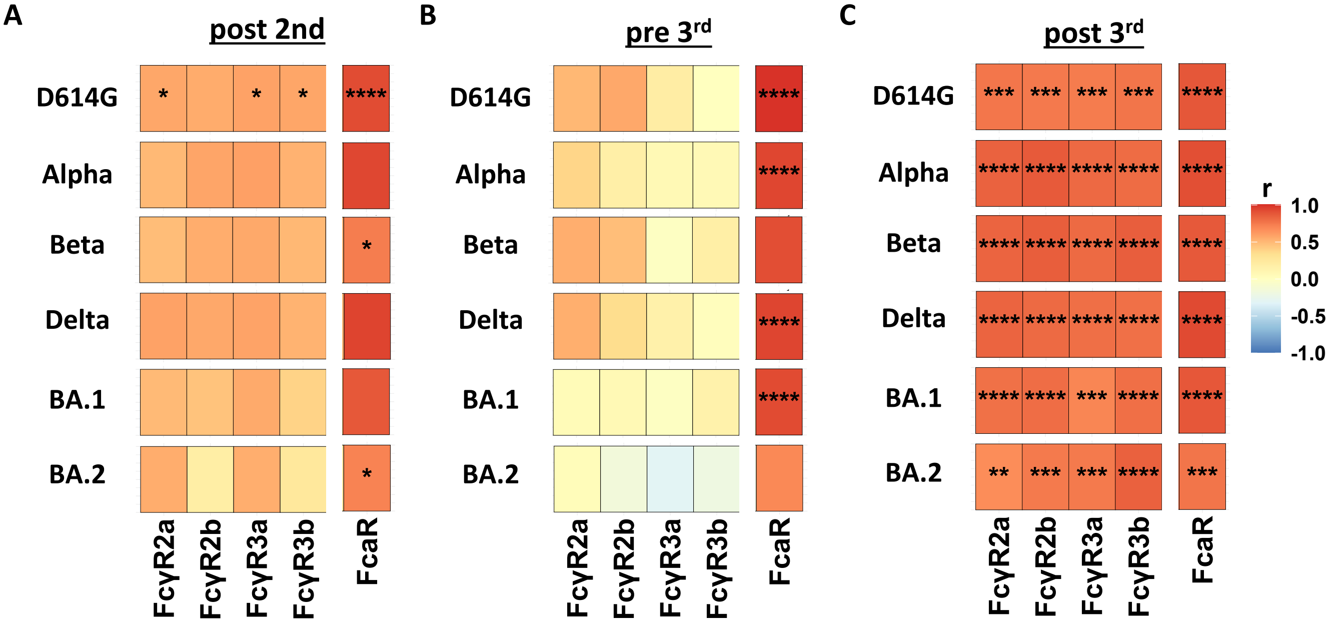

Figure S4. VOC-specific antibodies are highly correlated to Fc receptor binding. Related to Figure 2. The heatmap shows the spearman correlation coefficient of VOC IgG1 titers to the respective VOC FcγR or IgA1 to the FcαR in BNT162b2 vaccinated individuals at A) approx. 2 weeks after the second dose (post 2nd, n=18), B) before the third dose approx. 8 month after the second dose (pre 3rd, n=14) and C) approx. 2 weeks after the third dose (post 3rd, n=22). Color scale indicates the spearman correlation coefficient (only positive correlations were observed wit r>0) and asterisks indicate statistically significance of the correlation after Benjamini-Hochberg correction for multiple testing (**:p<0.01, ***:p<0.001, ****:p<0.0001).

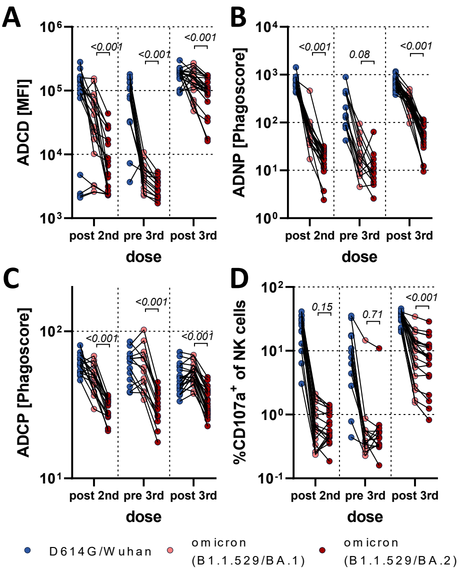

Figure S5. Fc functionality of BNT162b2 induced antibodies is superior to BA.1 compared to BA.2 antigen. Data as in Figure 3. Lines connect data from same individuals.

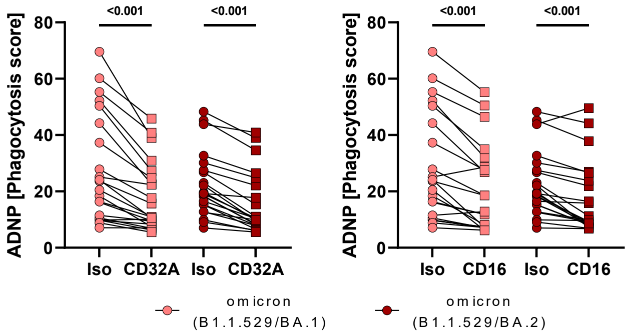

Figure S6. Role of FcγR2a (CD32A) and FcγR3 (CD16) on BA.1 and BA.2 specific ADNP. Related to Figure 3. Primary neutrophils were incubated with FcγR2a (CD32A, clone: IV.3, left panel) or FcγR3 (CD16, clone: LNK16, right panel) or respective isotype control (Iso) prior to ADNP. ADNP activity of BA.1 (light red) or BA.2 (dark red) specific antibodies of BNT162b2 vaccinated individuals (n=22) at the post-boost timepoint was analyzed.

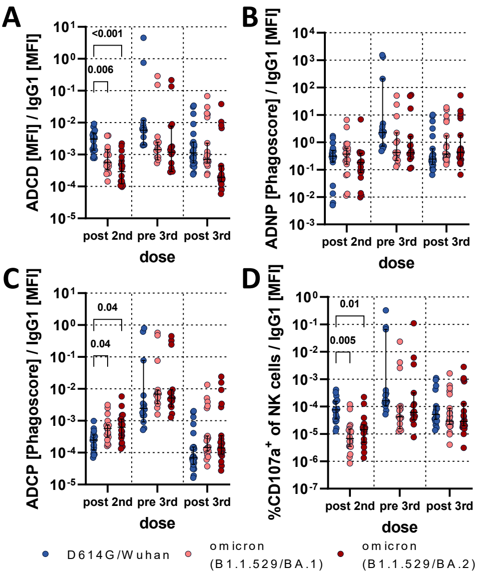

Figure S7. Fc functionality of BNT162b2 induced antibodies is superior to BA.1 compared to BA.2 antigen. Fc functions shown in Figure 3 were normalized for respective VOC IgG1 titer. Normalized Antibody-Dependent Complement Deposition (ADCD) (A), Neutrophil Phagocytosis (ADNP)(B), Cellular monocyte Phagocytosis (ADCP)(C), or NK cell activation (marked by CD107a expression)(D) of D614G, Omicron (B1.1.529) BA.1 or BA.2 specific antibodies is shown for BNT162b2 recipients at approx. 2 weeks after the second dose (post 2nd, n=18), before the third dose approx. 8 month after the second dose (pre 3rd, n=14) and approx. 2 weeks after the third dose (post 3rd, n=21). A Two-way-ANOVA with Benjamini-Hochberg post-test correction for multiple comparisons was used to test for statistical differences between D614G, BA1 and BA2 titers per timepoint, respectively. P-values are shown above each dataset. Horizontal lines indicate median and error bars the 95% confidence interval.

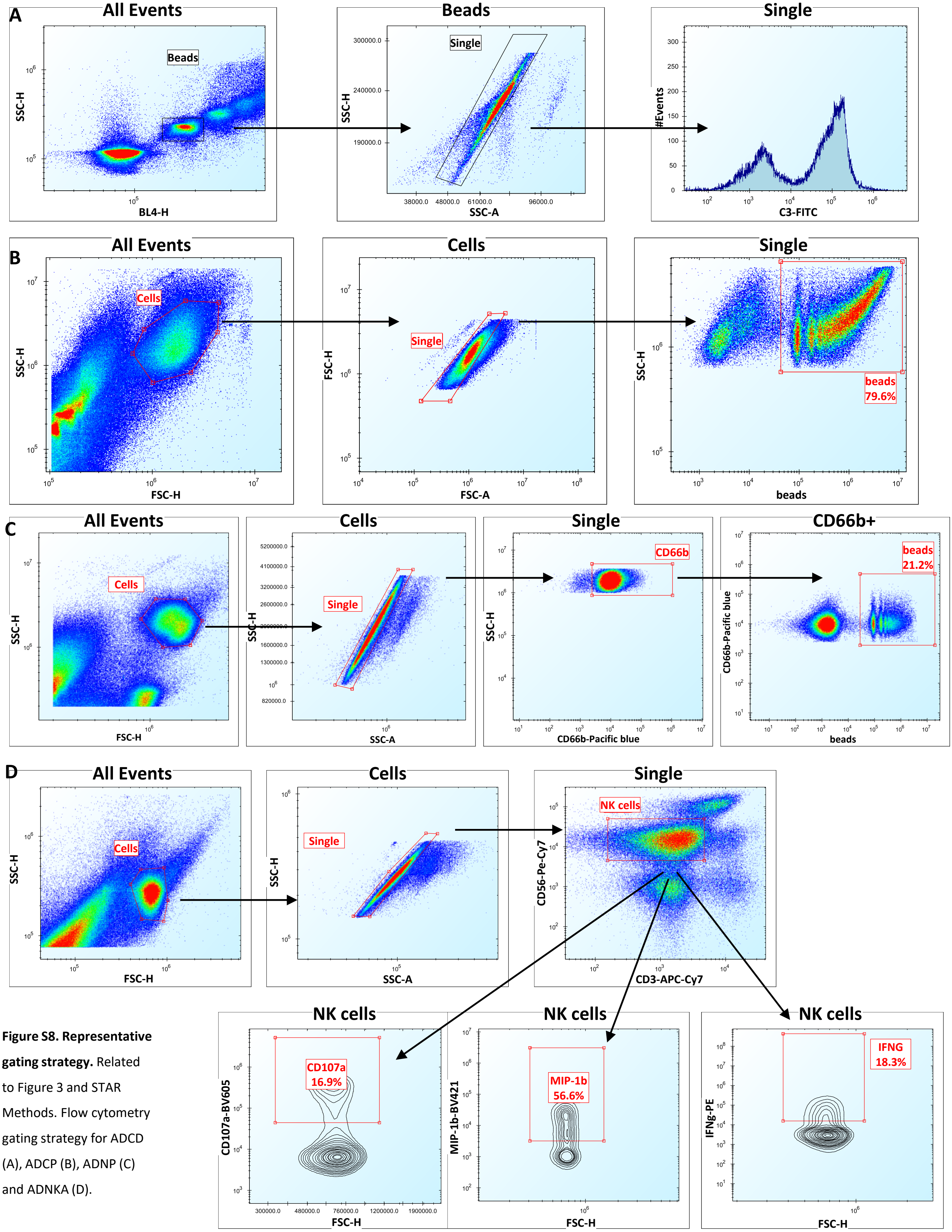

Figure S8. Representative gating strategy. Related to Figure 3 and STAR Methods. Flow cytometry gating strategy for ADCD (A), ADCP (B), ADNP (C) and ADNKA (D).
